# Biochemical phenotyping of paroxysmal nocturnal hemoglobinuria reveals solute carriers and β-oxidation deficiencies

**DOI:** 10.1371/journal.pone.0289285

**Published:** 2023-08-01

**Authors:** Patricia Eiko Yamakawa, Ana Rita Fonseca, Ismael Dale Cotrim Guerreiro da Silva, Matheus Vescovi Gonçalves, Dirce Maria Marchioni, Antonio Augusto Ferreira Carioca, David Michonneau, Celso Arrais-Rodrigues

**Affiliations:** 1 Hematology Division, Universidade Federal de São Paulo, São Paulo, Brazil; 2 Oncology Department, Hospital Sírio Libanês, São Paulo, Brazil; 3 Gynecology Department, Universidade Federal de São Paulo, São Paulo, Brazil; 4 Nutrition Department, School of Public Health, Faculdade de Medicina da Universidade de São Paulo, São Paulo, Brazil; 5 Nutrition Department, University of Fortaleza, Fortaleza, Brazil; 6 Hematology and Bone Marrow Transplant Department of the Saint-Louis Hospital, Paris, France; 7 Hematology Department, Hospital Nove de Julho, DASA, São Paulo, Brazil; Sohag University Faculty of Medicine, EGYPT

## Abstract

**Introduction:**

Paroxysmal nocturnal hemoglobinuria (PNH) is a clonal disease of hematopoietic cells with a variable clinical spectrum characterized by intravascular hemolysis, high risk of thrombosis, and cytopenias. To understand the biochemical shifts underlying PNH, this study aimed to search for the dysfunctional pathways involved in PNH physiopathology by comparing the systemic metabolic profiles of affected patients to healthy controls and the metabolomic profiles before and after the administration of eculizumab in PNH patients undergoing treatment.

**Methods:**

Plasma metabolic profiles, comprising 186 specific annotated metabolites, were quantified using targeted quantitative electrospray ionization tandem mass spectrometry in 23 PNH patients and 166 population-based controls. In addition, samples from 12 PNH patients on regular eculizumab maintenance therapy collected before and 24 hours after eculizumab infusion were also analyzed.

**Results:**

In the PNH group, levels of the long-chain acylcarnitines metabolites were significantly higher as compared to the controls, while levels of histidine, taurine, glutamate, glutamine, aspartate and phosphatidylcholines were significantly lower in the PNH group. These differences suggest altered acylcarnitine balance, reduction in the amino acids participating in the glycogenesis pathway and impaired glutaminolysis. In 12 PNH patients who were receiving regular eculizumab therapy, the concentrations of acylcarnitine C6:1, the C14:1/C6 ratio (reflecting the impaired action of the medium-chain acyl-Co A dehydrogenase), and the C4/C6 ratio (reflecting the impaired action of short-chain acyl-Co A dehydrogenase) were significantly reduced immediately before eculizumab infusion, revealing impairments in the Acyl CoA metabolism, and reached levels similar to those in the healthy controls 24 hours after infusion.

**Conclusions:**

We demonstrated significant differences in the metabolomes of the PNH patients compared to healthy controls. Eculizumab infusion seemed to improve deficiencies in the acyl CoA metabolism and may have a role in the mitochondrial oxidative process of long and medium-chain fatty acids, reducing oxidative stress, and inflammation.

## Introduction

Paroxysmal nocturnal hemoglobinuria (PNH) is a disorder caused by an acquired mutation in the phosphatidylinositol glycan class A (*PIG-A*) gene [1]. This leads to a partial or complete absence of all the GPI-linked proteins, including the complement regulatory proteins CD59 and CD55 [2], which results in an increased sensitivity of the red blood cells to the action of complement [3]. The clinical manifestations of PNH include hemolytic anemia, a high risk of thrombosis, and decreased hematopoiesis. The main cause of morbidity and mortality are thrombotic manifestations, which are common in atypical sites, such as in the hepatic venous system (Budd-Chiari syndrome); other causes include complications related to bone marrow failure, such as bleeding and infections [4].

Chronic intravascular hemolysis, the main hallmark for the clinical manifestations of PNH, is mediated by the alternative complement pathway. Normal red blood cells are protected against cytolysis mediated by the alternative complement pathway, mainly by CD55 and CD59 [5]. Deficiency of CD55 and CD59 in the erythrocytes of patients with PNH is the pathophysiological basis of intravascular hemolysis.

Eculizumab, a monoclonal antibody that binds to complement factor C5 [6], is effective in preventing its activation in C5b, inhibiting the formation of the membrane attack complex and changed the natural progression of the disease [7]. Patients treated with eculizumab showed a rapid improvement in hemolytic anemia with a significant decrease in lactate dehydrogenase (LDH) levels, a reduction in their need for transfusion, an improvement of nitric oxide (NO) depletion-related symptoms [8], and a consequent strong positive impact on their quality of life [9]. The use of eculizumab also appears to play a role in preventing both thrombosis progression and new thrombotic events [10, 11].

In clinical practice, some patients have suboptimal hematologic response to eculizumab, which present as persistence of anemia, maintenance of reticulocytosis, and biochemical evidence of hemolysis [12]. This phenomenon seems to be related to an extravascular hemolysis mechanism, which has not been completely elucidated yet. Patients treated with eculizumab have complement pathway blockage at the C5 level, but the early steps of the cascade, including activation, deposition, and proteolytic cleavage of C3 are unaffected, leading to excess deposition of C3 fragments in red blood cells and being recognized by macrophages present in the spleen and liver. Thus, this binding of C3 to red blood cells may constitute an additional mechanism of hemolysis in PNH, which cannot be prevented by treatment with eculizumab and produces a variable degree of extravascular hemolysis [13].

The metabolomic profile is able to provide a more precise functional measure of a phenotype formed as a result of genomic, transcriptomic, and proteomic changes [14]. Currently, the metabolomic study consists of the identification and quantification of small molecules in biological fluids (blood, urine, or tissues) and has been evaluated in many diseases, such as cancer and metabolic, infectious, and autoimmune diseases; it serves as a tool to better understand the biochemical state and possibly as biomarkers of diseases and clinical conditions [15, 16]. There are some studies that reports altered metabolomic profiles in hemolytic anemias other than PNH. Changes in the metabolites produced by endogenous glycolysis, endogenous glutathione and ascorbate metabolisms, membrane turnover, NO metabolism, and elevated sphingosine-1-phosphate were found in sickle cell erythrocytes [17–19]. Metabolomic analysis in β-thalassemia patients revealed alterations in multiple pathways including glycolysis, pyruvate, propanoate, glycerophospholipid, galactose, fatty acid, starch, and sucrose metabolism along with fatty acid elongation in mitochondria, glycerolipid, glyoxylate, and dicarboxylate metabolism [20]. Increase in aminoacids reflecting disturbances in membrane transporters were reported in aplastic anemia patients [21].

There are a few descriptions of metabolomics in patients with PNH [22]. The evaluation of the metabolomic profile of patients with PNH might be useful for revealing disease-related biomarkers and altered metabolic pathways; it also may provide a better understanding of the different clinical manifestations and discrepancies in response to complement inhibition and guide novel therapeutic possibilities.

## Methods

In total, 189 (23 PNH cases/166 controls) plasma samples were prospectively collected and analyzed. The PNH patients were followed by our group in 2 reference centers (Federal University of São Paulo and Hospital Sirio Libanes), both located in the city of São Paulo, SP, Brazil. The controls were participants of the São Paulo Population-based Health Investigation Project (ISA 2008). All the methods were carried out in accordance with relevant guidelines and regulations.

All 23 patients had their PNH diagnosis confirmed by flow cytometry performed using monoclonal antibodies against GPI-linked surface antigens. The following markers were used for diagnosis and monitoring clone size: CD14 PE (BD, clone MOP9), CD157 (BD, clone BP3), CD33 APC (BD, clone P67.6), CD24 (Abnova, clone ALB9), CD45 perCP (BD, clone 2D1), CD59 (clone 282h19) and FLAER (Alexa 488, Cedarlane). Only patients with a PNH clone size larger than 20% in granulocytes and/or monocytes were included. This study was approved by the Research Ethics Committee of the Federal University of Sao Paulo and Research Ethics Committee of Hospital Sirio Libanes. All patients and healthy controls agreed to participate in the study and were included after signing a written informed consent form. We confirm that all the experimental protocols were in accordance with guidelines of the Declaration of Helsinki.

Data collection included detailed information on the symptoms and signs of hemolysis (abdominal pain, hemoglobinuria, dysphagia, and fatigue), venous and/or arterial thromboembolism, transfusions, hospitalizations, hepatic and renal functions, PNH clone size, and on the treatment received for PNH.

Peripheral blood samples (10 ml) were collected using a syringe in tubes containing EDTA. All the patients receiving eculizumab were on the maintenance phase every 2 weeks and had their samples collected immediately prior to the infusion of eculizumab. In the 12 patients who were regular receiving eculizumab for at least 3 months, we also collected samples 24 hours after infusion. All the patients were free from red blood cells transfusions for at least three months before inclusion.

The tubes were centrifuged at 800 x g (gravitational force) for 10 minutes at 4°C to separate the plasma. The plasma was then centrifuged again at 1600 x g for 10 minutes at 4°C. Plasma fraction samples were collected and stored at -80°C in cryotubes and sent for analysis. All the samples arrived frozen. The quality control samples were within the pre-defined tolerances of the method.

### Absolute quantification of metabolites

The experimental metabolomics measurement technique is described in detail by patent US 2007/0004044 (accessible online at https://www.freepatentsonline.com/20070004044.html). Absolute quantifications (μmol/L) of blood metabolites were achieved by targeted quantitative profiling of metabolites (p180 kit) by electrospray ionization (ESI) tandem mass spectrometry (MS/MS) using the plasma samples (n = 189), blinded to any phenotype information, on a centralized, independent, fee-for-service basis on the quantitative metabolomics platform from BIOCRATES Life Sciences AG, Innsbruck, Austria. Briefly, a targeted profiling scheme was used to quantitatively screen for fully annotated metabolites using multiple reaction monitoring, neutral loss and precursor ion scans. Quantification of the metabolite concentrations and quality control assessments were performed using the MetIQ software package (BIOCRATES Life Sciences AG, Innsbruck, Austria) in conformance with 21CFR (Code of Federal Regulations Part 11), which implies proof of reproducibility within a given error range (https://biocrates.com/).

### Metabolites panel

In total, 186 metabolites were quantified, being: 40 acylcarnitines (ACs), 21 amino acids (AAs), 19 biogenic amines (BA), the sum of hexoses (Hex), 76 phosphatidylcholines (PCs), 14 lyso-phosphatidylcholines (LPCs) and 15 sphingomyelins (SMs). The glycerophospholipids were further differentiated with respect to the presence of ester (a) and ether (e) bonds in the glycerol moiety, where two letters denote that two glycerol positions are bound to a fatty acid residue (aa = diacyl, ae = acyl-alkyl), while a single letter indicates the presence of a single fatty acid residue (a = acyl or e = alkyl) (https://biocrates.com/).

In parallel to individual metabolite quantifications, groups of metabolites related to specific functions were analyzed. Groups of AAs were computed by summing the levels of AA belonging to certain families or chemical structures depending on their functions such as the sum of: 1. essential amino acids (Essential AA), 2. non-essential amino acids (non-Essential AA), 3. glucogenic (Alanine+Glycine+Serine) amino acids (Gluc AA), 4. branched-chain (Leucine+Iloleucine+Valine) amino acids (BCAA), 5. Aromatic (Histidine+Tyrosine+Tryptophan+Phenylalanine)amino acids (Arom AA), 6. Glutaminolytic derivatives (Alanine+Aspartame+Glutamate), and the sum of total amino acids. Groups of acylcarnitines (AC), important to evaluate mitochondrial function, were also computed by summing the total AC, C2+C3, C16+C18, C16+C18:1 and C16-OH+C18:1-OH/C12.

Groups of lipids, which are important to evaluate lipid metabolism, were also analyzed by summing: 1. Total lysophosphatidylcholines (total LPC), 2. Total acyl-acyl and 3. Total acyl-alkyl phosphatidylcholines (total PC aa and total PC ae, respectively), 4. Total sphingomyelins (total SM), and 5. Sum of total (LPC + PC aa +PC ae +SM) lipids (Structural lipids). A proxy for the alkylglyceronephosphate synthase (AGPS) activity was achieved by evaluating the proportions between the amount of acyl-alkyl phosphatidylcholines in relation to the sum of all phosphatidylcholine species detected using the p180 kit (Biocrates, Austria) [(https://biocrates.com/)].

Clinical indicators of liver metabolism and function were obtained by applying either the classic version (leucine+isoleucine+valine/(tyrosine+phenylalanine) or variations (Val/Phe, Xleu/Phe) of the Fischer`s quotient. Clinical indicators of isovaleric acidemia, tyrosinemia, urea cycle deficiency, and disorders of ß-oxidation were calculated by adopting the ratios of valerylcarnitine to butyrylcarnitine (C5/C4), tyrosine to serine (Tyr/Ser), glycine to alanine and glutamine (Gly/Ala, Gly/Gln), citrulline to ornithine (Cit/Orn), ornithine to arginine (Orn/Arg) [23–31]. Proxies for enzyme functions used in routine clinical practice were also utilized to aid in the diagnosis of methylmalonic and propionic acidemias as well as deficiencies of long (LCAD) and very long-chain acyl-CoA dehydrogenase (VLCAD) as well as type 2 carnitin-palmitoyl transferase (CPT-2) deficiencies: (C16-OH+C18:1-OH)/C12) (C16+C18:1/C2), (C14:1/C4), (C14:1-OH/C9), (C14/C9), (C14:1/C9), and the elongation of very-long-chain-fatty acids (ELOVL2) (PC aa C40:3/PC aa C42:5) [25, 32].

Of note, bearing in mind that proportions between the molar concentrations of metabolites are known to strengthen signal associations and, additionally, provide information for possible biochemical pathways we also scrutinized our samples through the utilization of 900 different ratios whose values can be used as proxies for enzyme functions for the following enzymes: fatty acid desaturase 1 (FADS1), short-chain acyl-coenzyme A dehydrogenase (ACADS), medium-chain acyl-coenzyme A dehydrogenase (ACADM), long-chain acyl-coenzyme A dehydrogenase (ACADL), elongation of very-long-chain fatty acids (ELOVL2), carbamoyl phosphate synthetase 1 (CPS1), protein-containing pleckstrin homology domain member 1 (PLEKHH1), spectrin repeat-containing nuclear envelope 2 (SYNE2), serine palmitoyltransferase long-chain base subunit 3 (SPTLC3), electron-transferring flavoprotein dehydrogenase (ETFDH), solute carrier family member 9 (SLC16A9), long-chain fatty acid coenzyme Aligase 1 (ACSL1), stearoyl coenzyme A sesaturase (SCD), 3-phosphoglycerate dehydrogenase (PHGDH), solute carrier family 22 (SLC22A4), apolipoprotein cluster (APO Cluster), glucokinase (Hexokinase 4) regulator (GCKR), melatonin receptor 1B (MTNR1B) [24, 30].

### Data analysis and validation tests

For metabolomic data analysis, log-transformations were applied to all quantified metabolites to normalize the concentration distributions and uploaded into the web-based analytical pipelines MetaboAnalyst 4.0 (www.metaboanalyst.ca) and Receiver Operating Characteristic Curve Explorer & Tester (ROCCET) available at http://www.roccet.ca/ROCCET for the generation of uni and multivariate Receiver Operating Characteristic (ROC) curves obtained through Support Vector Machine (SVM), Partial Least Squares-Discriminant Analysis (PLS-DA) and Random Forests as well as Logistic Regression Models to calculate Odds Ratios of specific metabolites.

ROC curves were generated by Monte-Carlo Cross Validation (MCCV) using balanced sub-sampling, where two thirds (2/3) of the samples were used to evaluate the feature importance. Significant features were then used to build classification models, which were validated on the 1/3 of the samples that were left out of the first analysis. The same procedure was repeated 10–100 times to calculate the performance and confidence interval of each model.

To further validate the statistical significance of each model, the ROC calculations included bootstrap 95% confidence intervals for the desired model specificity as well as accuracy after 1000 permutations and false discovery rates (FDR) calculations.

### Functional quantitative enrichment analysis

To fully exploit our data as well as further validate our hypothesis we also performed unsupervised “*in silico*” functional analysis by uploading the entire quantitative acylcarnitine set from the healthy and PNH-affected participants to the “Metabolite Set Enrichment Analysis (MSeA)” tool available at www.metaboanalyst.ca.

## Results

### Clinical features

Twenty-three PNH patients were enrolled in this study. The clinical and laboratorial characteristics of each patient are shown in Tables 1 and 2a and 2b. The median age was 31 years, ranging from 18 to 69 years, and 14 patients (61%) were male. Sixteen patients (70%) reported hemoglobinuria, 11 (48%) reported abdominal pain, and 20 (87%) had a history of transfusion requirements. Eight patients presented with thrombotic events, seven presented with venous thrombosis (five had abdominal vein thrombosis, including two Budd-Chiari syndrome), and one presented with arterial thrombosis (transient ischemic stroke). Most of the PNH patients (83%) were receiving anti-complement therapy with eculizumab in the maintenance phase, and the median time on medication was 41 months (range 3–84 months).

**Table 1:** Clinical characteristics of PNH patients.

Patient	Age	Sex	Thrombosis	Transfusion	Hemoglobinuria	Abdominal pain
**1**	**32**	**M**	**No**	**No**	**Yes**	**No**
**2**	**41**	**F**	**No**	**Yes**	**Yes**	**No**
**3**	**32**	**M**	**Mesenteric venous thrombosis**	**Yes**	**No**	**Yes**
**4**	**34**	**F**	**No**	**No**	**Yes**	**Yes**
**5**	**25**	**M**	**No**	**Yes**	**No**	**No**
**6**	**57**	**F**	**No**	**Yes**	**Yes**	**No**
**7**	**39**	**F**	**Lower extremity deep vein thrombosis**	**Yes**	**Yes**	**Yes**
**8**	**61**	**F**	**No**	**Yes**	**Yes**	**No**
**9**	**27**	**M**	**No**	**Yes**	**Yes**	**Yes**
**10**	**30**	**M**	**No**	**Yes**	**Yes**	**No**
**11**	**39**	**F**	**Cerebral venous thrombosis, mesenteric and splenic venous thrombosis**	**Yes**	**Yes**	**Yes**
**12**	**52**	**M**	**No**	**No**	**No**	**No**
**13**	**28**	**M**	**No**	**Yes**	**No**	**No**
**14**	**57**	**M**	**Lower extremity deep vein thrombosis** **Splenic vein thrombosis**	**Yes**	**Yes**	**Yes**
**15**	**37**	**M**	**No**	**Yes**	**Yes**	**Yes**
**16**	**72**	**M**	**No**	**Yes**	**No**	**No**
**17**	**36**	**M**	**No**	**Yes**	**No**	**No**
**18**	**40**	**M**	**Budd Chiari, Acute ischemic stroke**	**Yes**	**Yes**	**Yes**
**19**	**50**	**F**	**Transient ischemic attack**	**Yes**	**Yes**	**No**
**20**	**68**	**F**	**Budd Chiari, Acute ischemic stroke**	**Yes**	**Yes**	**Yes**
**21**	**64**	**F**	**No**	**Yes**	**Yes**	**No**
**22**	**30**	**M**	**Lower extremity deep vein thrombosis**	**Yes**	**No**	**Yes**
**23**	**36**	**M**	**No**	**Yes**	**Yes**	**Yes**

**Table 2:** Laboratorial characteristics of PNH patients.

**a**											
**Patient**	**Eculizumab**	**Hemoglobin at diagnosis (12–15,5 g/dL)**	**Hemoglobin after treatment**	**LDH at diagnosis (ULN 250–480 U/L)**	**LDH after treatment**	**Reticulocytes at diagnosis (30000-100000/mm3)**	**Reticulocytes after treatment**	**Leucocytes at diagnosis (3500-10500/mm3)**	**Leucocytes after treatment**	**Platelets at diagnosis (150000–450000/mm3)**	**Platelets after treatment**
**1**	**Yes**	**6,6**	**12**	**2277 (4,74 x ULN)**	**474 (0,98 x ULN)**	**224130**	**279700**	**4290**	**4290**	**208000**	**137000**
**2**	**Yes**	**4,7**	**9,9**	**2812 (5,85 x ULN)**	**520 (1,08 x ULN)**	**154600**	**300000**	**3340**	**4420**	**67000**	**113000**
**3**	**Yes**	**9,4**	**12,8**	**1455 (3,03 x ULN)**	**387 (0,8 x ULN)**	**133000**	**113000**	**6700**	**3720**	**49000**	**95000**
**4**	**Yes**	**10,7**	**10,5**	**1891 (3,93 x ULN)**	**624 (1,3 x ULN)**	**107840**	**200700**	**4380**	**5670**	**78000**	**177000**
**5**	**Yes**	**5,9**	**11**	**1603 (3,33 x ULN)**	**493 (1,02 x ULN)**	**161000**	**181700**	**3200**	**2700**	**36000**	**102000**
**6**	**Yes**	**9,2**	**9,9**	**3989 (8,31 x ULN)**	**379 (0,78 x ULN)**	**336000**	**272100**	**3330**	**2720**	**152000**	**158000**
**7**	**Yes**	**9,1**	**10**	**3720 (7,75 x ULN)**	**277 (0,57 x ULN)**	**163360**	**156480**	**9160**	**6560**	**213000**	**232000**
**8**	**Yes**	**11,1**	**8,1**	**978 (2,03 x ULN)**	**539 (1,12 x ULN)**	**804000**	**669200**	**5500**	**5020**	**153000**	**132000**
**9**	**Yes**	**9,1**	**8,7**	**2133 (4,44 x ULN)**	**435 (0,9 x ULN)**	**420000**	**342100**	**7867**	**3710**	**67000**	**116000**
**10**	**Yes**	**8,1**	**11,5**	**3323 (6,92 x ULN)**	**612 (1,27 x ULN)**	**153300**	**91100**	**1510**	**2000**	**74000**	**106000**
**11**	**Yes**	**8,7**	**8,1**	**773 (3,09xULN)**	**442 (1,76 x ULN)**	**162700**	**120100**	**4730**	**1400**	**137000**	**159000**
**12**	**Yes**	**13,5**	**11,7**	**492 (1,96 x ULN)**	**372 (1,48 x ULN)**	**396000**	**189500**	**4730**	**3950**	**110000**	**185000**
**13**	**Yes**	**11,5**	**11,5**	**1662 (2,42 x ULN)**	**718 (1,49 x ULN)**	**190000**	**169000**	**3380**	**3420**	**63000**	**97000**
**14**	**Yes**	**9,6**	**13,5**	**2586 (5,38 x ULN)**	**369 (0,76 x ULN)**	**420000**	**132900**	**9440**	**3100**	**141000**	**198000**
**15**	**Yes**	**12,2**	**12,7**	**3783 (7,88 x ULN)**	**449 (0,93 x ULN)**	**213400**	**424800**	**4460**	**4280**	**157000**	**182000**
**16**	**No**	**7,8**	**-**	**1691 (3,52 x ULN)**	**-**	**157200**	**-**	**4370**	**-**	**8000**	.
**17**	**No**	**7,8**	**-**	**888 (1,85 x ULN)**	**-**	**190000**	**-**	**2200**	**-**	**48000**	.
**18**	**No**	**9,7**	**-**	**1836 (3,82 x ULN)**	**-**	**186000**	**-**	**3990**	**-**	**129000**	.
**19**	**Yes**	**8,5**	**8,7**	**1760 (3,66 x ULN)**	**458 (0,95 x ULN)**	**132000**	**250440**	**5100**	**3230**	**185000**	**242000**
**20**	**Yes**	**6,6**	**13,5**	**2504 (5,21 x ULN)**	**486 (1,01 x ULN)**	**190300**	**71600**	**2770**	**8100**	**23000**	**105000**
**21**	**Yes**	**7,8**	**7,2**	**520 (2,08 x ULN)**	**411 (1,64 x ULN)**	**152000**	**59000**	**3000**	**3300**	**89000**	**105000**
**22**	**No**	**7,6**	**-**	**2078 (4,32 x ULN)**	**-**	**170000**	.	**4580**	.	**85000**	.
**23**	**Yes**	**13,6**	**12**	**407 (1,62 x ULN)**	**223 (0,89 x ULN)**	**130000**	**80000**	**5730**	**4160**	**110000**	**163000**
**b**											
**Patient**	**Eculizumab**	**% Erythrocyte PNH clone at diagnosis**		**% Erythrocyte PNH clone after treatment**	**% Granulocytes PNH clone at diagnosis**	**% Granulocytes PNH clone after treatment**		**% Monocytes PNH clone at diagnosis**		**% Monocytes PNH clone after treatment**	
**1**	**Yes**	**95**		**78**	**90**	**99,8**		**90**		**99,5**	
**2**	**Yes**	**27**		**74**	**92**	**99**		**79**		**98**	
**3**	**Yes**	**20**		**8,50**	**70**	**98**		**100**		**98**	
**4**	**Yes**	**30**		**37**	**70**	**71**		**81**		**74**	
**5**	**Yes**	**6**		**78,90**	**55**	**62,7**		**98**		**95,4**	
**6**	**Yes**	**90**		**95**	**99,56**	**97**		**99,86**		**99,7**	
**7**	**Yes**	**63**		**40**	**73,81**	**55**		**90,5**		**45**	
**8**	**Yes**	**50**		**76**	**70**	**99,5**		**98**		**99,5**	
**9**	**Yes**	**30**		**87**	**84**	**99**		**86**		**99**	
**10**	**Yes**	**51**		**57,90**	**99**	**90**		**93**		**90**	
**11**	**Yes**	**58**		**42**	**96**	**88**		**96,5**		**68**	
**12**	**Yes**	**20**		**24**	**66,5**	**74,5**		**87**		**72,5**	
**13**	**Yes**	**30**		**60**	**65**	**63**		**63**		**70**	
**14**	**Yes**	**51**		**12**	**82**	**18**		**92**		**32**	
**15**	**Yes**	**42**		**53**	**90**	**94**		**80**		**94**	
**16**	**No**	**49**		**-**	**19,6**	**-**		**21**		**-**	
**17**	**No**	**10**		**-**	**15**	**-**		**29,6**		**-**	
**18**	**No**	**86**		**-**	**89**	**-**		**15**		**-**	
**19**	**Yes**	**23,00**		**33**	**79**	**89**		**88**		**89**	
**20**	**Yes**	**66,00**		**11**	**56**	**30**		**88**		**33**	
**21**	**Yes**	**67,00**		**26**	**36**	**99**		**52**		**99**	
**22**	**No**	**31**		**-**	**70**	**-**		**49**		**-**	
**23**	**Yes**	**29**		**64**	**95**	**97,9**		**99**		**98,9**	

Hb: hemoglobin, LDH: lactate dehydrogenase, ULN: upper limit of normal

### PNH metabolic profile

In the patients with hemolytic PNH, 92 out of the 186 metabolites analyzed had significantly different concentrations in the PNH patients compared to the controls, with positive or negative correlations (MetaboAnalyst 4.0) (Table 3).

**Table 3:** Pearson r correlation results.

Metabolites	Correlation	T-Stat	p- value	FDR
**C6:1**	0,81	18,72	1,21E-44	1,04E-42
**C16-OH**	0,48	7,47	3,08E-12	8,80E-11
**C18**	0,44	6,70	2,38E-10	5,87E-09
**C18:1**	0,44	6,37	1,41E-09	2,71E-08
**C16**	0,41	6,07	7,05E-09	1,11E-07
**C3-OH**	0,38	5,56	9,48E-08	1,07E-06
**C162**	0,38	5,53	0,0010	0,0012
**Spermidine**	0,42	5,41	2,69E-07	3,35E-06
**C16:1**	0,37	5,38	2,18E-07	0,0002
**PC aa C30:2**	0,37	4,70	6,15E-06	5,94E-05
**C18:2**	0,32	4,65	6,26E-06	4,17E-05
**C12-DC**	0,34	4,28	3,45E-05	0,0003
**PC aa C26:0**	0,26	3,16	0,0019	0,0065
**Ornithine**	0,25	2,98	0,0034	0,0106
**lysoPC a C26:1**	0,23	2,78	0,0062	0,0176
**C3**	0,20	2,44	0,0159	0,0365
**PC ae C30:0**	0,20	2,42	0,0166	0,0370
**PC ae C30:1**	0,20	2,35	0,0202	0,0413
**Asparagine**	0,17	2,04	0,0431	0,0824
**lysoPC a C26:0**	0,17	2,01	0,0462	0,0874
**PC ae C36:5**	-0,17	-2,04	0,0429	0,0824
**PC ae C42:5**	-0,18	-2,10	0,0379	0,0741
**PC aa C32:1**	-0,18	-2,20	0,0298	0,0589
**PC ae C36:2**	-0,19	-2,27	0,0245	0,0490
**PC aa C42:5**	-0,19	-2,32	0,0217	0,0440
**PC aa C38:4**	-0,20	-2,35	0,0201	0,0413
**Methionine**	-0,20	-2,37	0,0191	0,0400
**lysoPC a C24:0**	-0,20	-2,37	0,0190	0,0400
**PC aa C42:6**	-0,20	-2,41	0,0173	0,0372
**PC ae C38:0**	-0,20	-2,41	0,0171	0,0372
**PC ae C38:1**	-0,20	-2,42	0,0168	0,0370
**Serine**	-0,20	-2,43	0,0163	0,0368
**C14:1-OH**	-0,21	-2,47	0,0147	0,0340
**PC ae C44:5**	-0,21	-2,48	0,0142	0,0334
**C12**	-0,21	-2,49	0,0140	0,0334
**PC ae C42:1**	-0,21	-2,51	0,0131	0,0317
**PC aa C36:5**	-0,21	-2,54	0,0120	0,0295
**Leucine**	-0,22	-2,64	0,0092	0,0228
**PC ae C34:3**	-0,22	-2,65	0,0089	0,0225
**PC aa C42:1**	-0,22	-2,67	0,0085	0,0218
**C14**	-0,22	-2,68	0,0084	0,0217
**PC ae C40:2**	-0,22	-2,69	0,0080	0,0212
**PC ae C36:0**	-0,23	-2,74	0,0069	0,0184
**PC aa C38:5**	-0,23	-2,75	0,0067	0,0182
**PC aa C32:3**	-0,23	-2,76	0,0065	0,0179
**PC ae C42:0**	-0,23	-2,77	0,0063	0,0177
**C102**	-0,23	-2,78	0,0061	0,0176
**lysoPC a C18:0**	-0,23	-2,80	0,0058	0,0171
**PC ae C40:1**	-0,23	-2,81	0,0057	0,0171
**PC aa C34:4**	-0,24	-2,86	0,0050	0,0151
**PC aa C38:3**	-0,24	-2,93	0,0040	0,0125
**PC aa C34:1**	-0,25	-2,98	0,0034	0,0106
**PC aa C36:6**	-0,25	-2,99	0,0033	0,0106
**PC ae C44:3**	-0,25	-3,10	0,0024	0,0079
**PC ae C44:6**	-0,26	-3,18	0,0018	0,0063
**PC aa C38:6**	-0,26	-3,22	0,0016	0,0057
**PC aa C42:0**	-0,26	-3,23	0,0015	0,0056
**Arginine**	-0,27	-3,24	0,0015	0,0055
**PC aa C40:2**	-0,27	-3,24	0,0015	0,0055
**PC aa C34:3**	-0,27	-3,26	0,0014	0,0054
**PC aa C36:4**	-0,27	-3,28	0,0013	0,0053
**Glutamine**	-0,27	-3,28	0,0013	0,0053
**PC ae C36:1**	-0,27	-3,28	0,0013	0,0053
**Alanine**	-0,27	-3,29	0,0013	0,0053
**PC aa C42:2**	-0,27	-3,30	0,0012	0,0053
**PC aa C36:0**	-0,27	-3,30	0,0012	0,0053
**PC ae C38:3**	-0,27	-3,34	0,0011	0,0050
**C6 C41-DC**	-0,28	-3,37	0,0010	0,0046
**Valine**	-0,28	-3,41	0,0008	0,0042
**PC aa C32:2**	-0,28	-3,44	0,0008	0,0039
**lysoPC a C20:3**	-0,28	-3,47	0,0007	0,0036
**PC ae C38:2**	-0,29	-3,54	0,0005	0,0030
**C0**	-0,31	-3,78	0,0002	0,0013
**PC aa C40:1**	-0,31	-3,80	0,0002	0,0013
**PC ae C42:3**	-0,31	-3,89	0,0002	0,0010
**PC aa C36:2**	-0,31	-3,90	0,0001	0,0010
**C18:1**	-0,33	-4,09	7,32E-05	0,0005
**PC aa C36:3**	-0,30	-4,34	2,31E-05	0,0001
**Histidine**	-0,32	-4,56	9,17E-06	0,0006
**C14:2**	-0,32	-4,68	5,42E-06	0,0004
**PC aa C34:2**	-0,33	-4,70	5,08E-06	4,13E-05
**C14:1**	-0,33	-4,80	3,18E-06	2,75E-05
**Glutamate**	-0,34	-4,95	0,0001	0,0018
**PC ae C40:3**	-0,39	-5,01	1,62E-06	1,88E-05
**PC aa C40:3**	-0,40	-5,95	1,32E-08	0,0001
**Taurine**	-0,40	-6,00	1,01E-08	1,45E-07
**Aspartate**	-0,41	-6,16	4,49E-09	7,76E-08
**C142-OH**	-0,44	-6,61	4,09E-10	8,85E-09
**C10:1**	-0,54	-8,77	1,13E-15	3,90E-14
**C12:1**	-0,55	-8,90	4,90E-16	2,12E-14
**C16:2-OH**	-0,93	-34,44	1,02E-82	2,08E-80
**C6**	-0,71	-34,44	1,20E-82	2,08E-80

Single metabolites detected in blood whose concentrations were positively (Salmon) or negatively (Blue) correlated to PNH compared to the controls. In the first column are the groups of metabolites arranged from the highest percentage and significance of correlation with PNH separated in color according to their correlation (salmon rows: increased metabolites in the PNH group; blue rows: reduced metabolites in the group with PNH). In the second column, a positive correlation with PNH was demonstrated in decimal and non-percentage values; a negative correlation with PNH has the minus (-) sign. In the third column, is the p-values (considered statistically significant when p <0.05). In the fourth column, is the FDR: the percentages indicate the expected false positives among all the predicted characteristics as significant; this excludes false positives and increases the significance of the findings.

**Fig 1:**
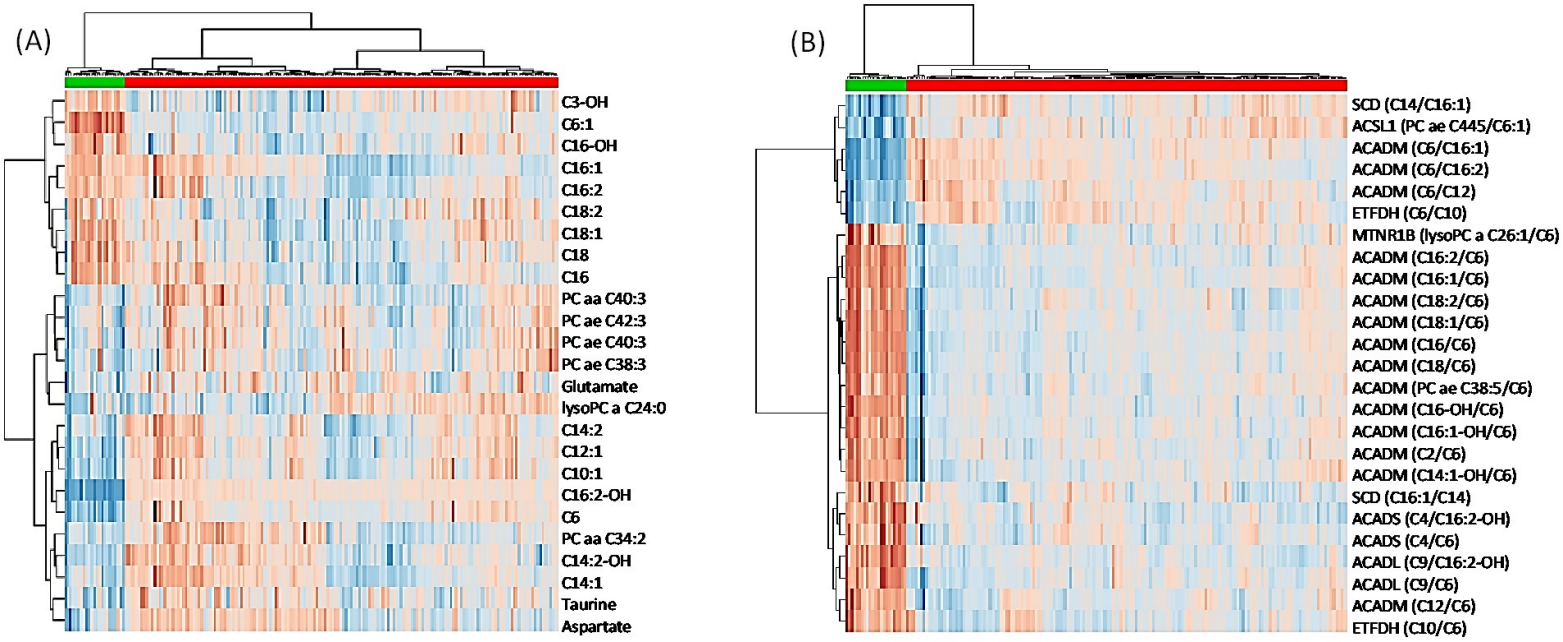
Unsupervised multivariate clustering. Fig 1A shows the top 25 single metabolites (A) detected in blood whose concentrations are elevated (Red) or decreased (Blue) when comparing PNH cases to controls. In Fig 1B, the single metabolites are now assembled as specific ratios whose values can be used as proxies for gene function. Notably, the utilization of properly assembled ratios is known to strengthen signal associations with consequent increases in the statistical significance. Abbreviations: ACADS = acyl-Coenzyme A Dehydrogenase Short Chain, ACADM = acyl-Coenzyme A Dehydrogenase Medium Chain, ACADL = acyl-Coenzyme A Dehydrogenase Long Chain, ETFDH = Electron-transferring Flavoprotein Dehydrogenase, ACSL1 = Long-chain Fatty Acid Coenzyme A Ligase 1, SCD = Stearoyl Coenzyme A Desaturase. (Metaboanalyst 4.0 www.metaboanalyst.ca).Published at https://doi.org/10.1182/blood-2019-126241.

A heatmap (Fig 1a and 1b) was created to identify the pattern and trend of distribution of the metabolites in the PNH patients (upper green line) and in controls (upper red line).

**Fig 2:**
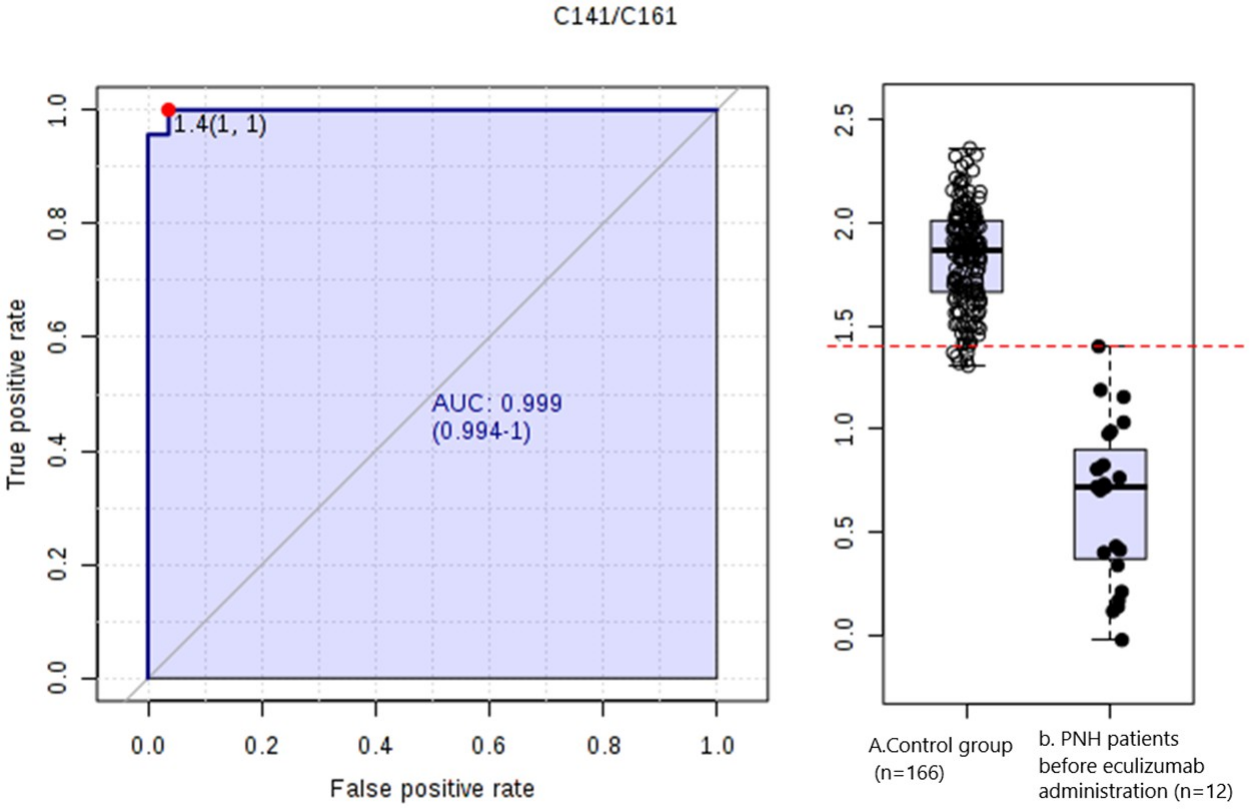
C14:1/C16:1 ratio of the PNH patients before eculizumab administration. ROC curve of the C14:1/C16:1 ratio, with cutoff values based on area under the curve (AUC), 95% CI: cutoff 1.4; sensitivity 0.95 (0.87–1); specificity 0.96 (0.93–0.99); positive likelihood ratio 27.66; negative likelihood ratio 0,0. The y-axis corresponds to the fraction of true positives (equivalent to the sensitivity of the method tested), and the x-axis corresponds to the fraction of false positives (equivalent to the specificity). The plot point in the upper corner (upper left) is the ideal point of the exam (sensitivity and specificity close to 100%). The C14:1/C16:1 ratio has lower values in the PNH patients compared to the control group.

The metabolites with significantly altered concentrations in the PNH patients are described in Tables 3 and 4. Several combinations of pairs of metabolites found in the PNH patients were tested. The ratio of C14:1/C16:1 acylcarnitines (tetradecenylcarnitine / palmitoleylcarnitine) resulted in the highest statistical significance, demonstrating a 95% sensitivity and 96% specificity (ROC curve—Fig 2) for values lower than 1.4.

**Table 4:** The metabolites are combined as specific ratios whose values can be used as proxies for gene function.

Gene	Ratios	Correlation	T-Stat	p-value	FDR
**ACADM**	(C16:2/C6)	0.85	21.95	1.65E-53	7.14E-51
**ACADL**	(C9/C16:2-OH)	0.83	20.50	1.33E-49	2.89E-47
**ACADM**	(C16:1/C6)	0.81	18.85	5.19E-45	8.99E-43
**ACADM**	(C16/C6)	0.81	18.72	1.19E-44	1.71E-42
**ACADM**	(C18:1/C6)	0.79	17.69	1.04E-41	1.12E-39
**ACADM**	(C18/C6)	0.79	17.35	9.84E-41	9.47E-39
**ACADM**	(C16-OH/C6)	0.78	16.75	5.26E-39	4.55E-37
**ACADL**	(C9/C6)	0.75	15.41	4.68E-35	3.38E-33
**ACADS**	(C4/C16:2-OH)	0.75	15.32	8.65E-35	5.76E-33
**ACADM**	(C18:2/C6)	0.70	13.53	1.77E-29	1.02E-27
**ACADM**	(C16:1-OH/C6)	0.70	13.45	3.13E-29	1.69E-27
**SCD**	(C16:1/C14)	0.69	13.05	4.66E-28	2.37E-26
**ACADM**	(C12/C6)	0.69	12.97	8.28E-28	3.98E-26
**ACADM**	(C2/C6)	0.69	12.91	1.21E-27	5.23E-26
**ACADM**	(PC ae C38:5/C6)	0.67	12.45	2.92E-26	1.17E-24
**ACADM**	(C14:1-OH/C6)	0.67	12.39	4.43E-26	1.67E-24
**ACADS**	(C4/C6)	0.67	12.29	8.58E-26	3.10E-24
**ACADM**	(C6/C14)	-0.66	-12.07	3.87E-25	1.34E-23
**ETFDH**	(C10/C16:2)	-0.67	-12.44	2.98E-26	1.17E-24
**ETFDH**	(C6/C10)	-0.69	-12.94	1.02E-27	4.66E-26
**ACADM**	(C6/C12)	-0.74	-14.88	1.71E-33	1.06E-31
**ACSL1**	(PC ae C445/C6:1)	-0.76	-15.84	2.51E-36	1.98E-34
**SCD**	(C14/C16:1)	-0.79	-17.73	7.68E-42	9.51E-40
**ACADM**	(C6/C16:1)	-0.83	-20.66	5.01E-50	1.45E-47
**ACADM**	(C6/C16:2)	-0.87	-24.17	2.71E-59	2.34E-56

Notably, the utilization of properly assembled ratios is known to strengthen signal associations with consequent increases in statistical significance. This possibility can be analyzed by comparing the p-values and FDRs. Abbreviations: ACADS = acyl-Coenzyme A Dehydrogenase Short Chain, ACADM = acyl-Coenzyme A Dehydrogenase Medium Chain, ACADL = acyl-Coenzyme A Dehydrogenase Long Chain, ETFDH = Electron-transferring Flavoprotein Dehydrogenase, ACSL1 = Long-chain Fatty Acid Coenzyme A Ligase 1, SCD = Stearoyl Coenzyme A Desaturase.

**Fig 3:**
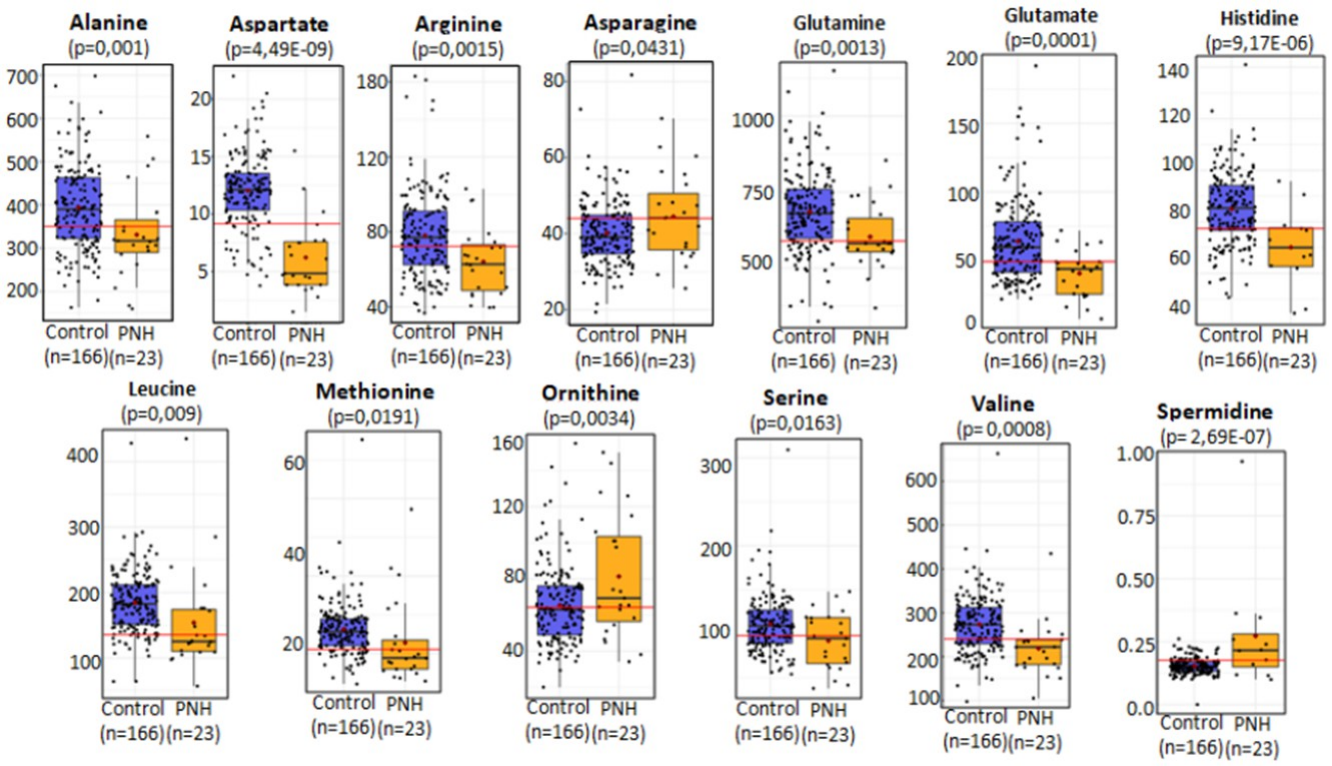
Amino acids levels in the PNH patients compared to controls. Alanine: 318 μM/L (160–559) vs 388 μM/L (163–675), Aspartate: 4,67 μM/L (0,1–15) vs 10,2 μM/L (3,74–22), Arginine: 62 μM/L (39–103) vs 77 μM/L (36–163), Asparagine: 44 μM/L (25–70) vs 38 μM/L (19–81), Glutamine: 578 μM/L (368–854) vs 683μM/L (324–1150), Glutamate: 44 μM/L (7–71) vs 59μM/L (21–192), Histidine: 73 μM/L (44–98) vs 85μM/L (50–141), Leucine: 124 μM/L (56–435) vs 181μM/L (63–428). Methionine: 18 μM/L (12–57) vs 25 μM/L (12–75), Ornithine: 67 μM/L (34–150) vs 63 μM/L (20–155), Serine: 96 μM/L (38–148) vs 110 μM/L (54–309), Valine: 217 μM/L (105–435) vs 269 μM/L (97–663), Spermidine: 0,26 μM/L (0–2,6) vs 0 μM/L (0–0,63) The PNH patients had lowers concentrations of alanine, aspartate, arginine, glutamine, glutamate, histidine, leucine, methionine, serine, and valine compared to the control group. Asparagine, ornitine, and spermidide were found in higher concentrations in the PNH patients compared to the control group.

From the structural lipids perspective, the PNH metabolic scenario was mainly characterized by lower levels of circulating structural, diacyl, and acyl-alkyl, phospholipids suggesting decreased biosynthesis or increased consumption. Indeed, the total amount of phosphatidylcholines was significantly decreased. Similarly, amino acids levels (Fig 3) alanine, aspartate, valine, glutamine, leucine, histidine, taurine, glutamate, serine, and methionine were found in lower concentrations when compared to the controls. Ornithine (p = 0.003) and spermidine (p = 2.69, E-07) molar levels were significantly elevated, in parallel to decreasing levels of arginine (p = 0.0015), making these combinations suggestive of urea cycle disorders and dysfunctions in biogenic amines metabolism.

**Fig 4:**
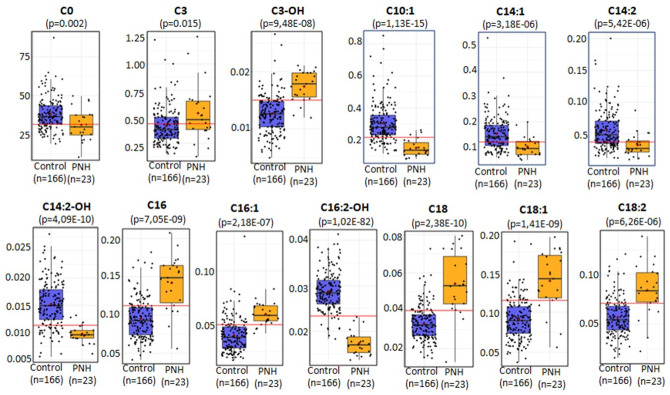
Acylcarnitines levels in the PNH patients compared to the controls. C0: 31 μM/L (10–49) vs 36 μM/L (19–87); C3: 0,49μM/L (0,12–1,25) vs 0,38 μM/L (0,15–1,22); C3-OH: 0,018 μM/L (0,01–0,02) vs 0,012 μM/L (0,005–0,02); C10:1: 0,14 μM/L (0,08–0,26) vs 0,27 μM/L (0,11–0,85), C14:1: 0,09 μM/L (0,04–0,19) vs 0,13 μM/L (0,05–0,53), C14:2: 0,03 μM/L (0,009–0,08) vs 0,05 μM/L (0,01–0,20), C14:2-OH: 0,009 μM/L (0,006–0,01) vs 0,01 μM/L (0,005–0,02), C16: 0,14 μM/L (0,05–0,20) vs 0,09 μM/L (0,04–0,18), C16:1: 0,05 μM/L (0,04–0,08) vs 0,03 μM/L (0,01–0,13), C16:2-OH: 0,017 μM/L (0,012–0,023) vs 0,028 μM/L (0,018–0,041), C18: 0,05 μM/L (0,012–0,079) vs 0,03 μM/L (0,014–0,072), C18:1: 0,13 μM/L (0,05–0,19) vs 0,08 μM/L (0,03–0,18), C18;2: 0,08 μM/L (0,02–0,13) vs 0,05 μM/L (0,01–0,12). The PNH patients had lowers concentrations of C0, C10:1, C14:1, C14:2, C14:2-OH, and C16:2-OH compared to the control group. C3, C3-OH, C16, C16:1, C18, C18;1, and C18:2 were found in in higher concentrations in the PNH patients compared to the control group.

Unsupervised quantitative metabolite clustering and Pearson r correlation analysis, revealed specific biochemical deviations characterized by significant elevations (C3, C3-OH, C16, C16:1, C18, C18:1, C18:2) and reductions (C0, C16:2-OH, C14:2-OH, C14:1, C14:2, C10:1) in the blood molar levels of acylcarnitines species (Fig 4). The mean plasma free L-carnitine concentration, in the PNH group, was significantly lower than its mean concentration level in the control group, 31 μM/L (10–49 μM/L) vs 38 μM/L (19–87 μM/L), respectively (p = 0.0002, FDR = 0.0013).

In order to more precisely gain access to specific candidate pathways, that could be deregulated in PNH, we assembled our metabolites according 900 specific ratios and used them as proxies for enzyme function [21, 28].

In total agreement with our hypothesis linking perturbations in acyl CoA metabolism to PNH, unsupervised quantitative clustering analysis, using the heatmap (Fig 1b), and the Pearson r correlation analysis revealed (with p Values of association <10e-25) that PNH patients share a significant degree of correlation to ACADS, ACADM, ACADL and ETFDH insufficiencies, as well as to ACSL1 and SCD (Table 4).

### Metabolic profile in patients on eculizumab

**Fig 5:**
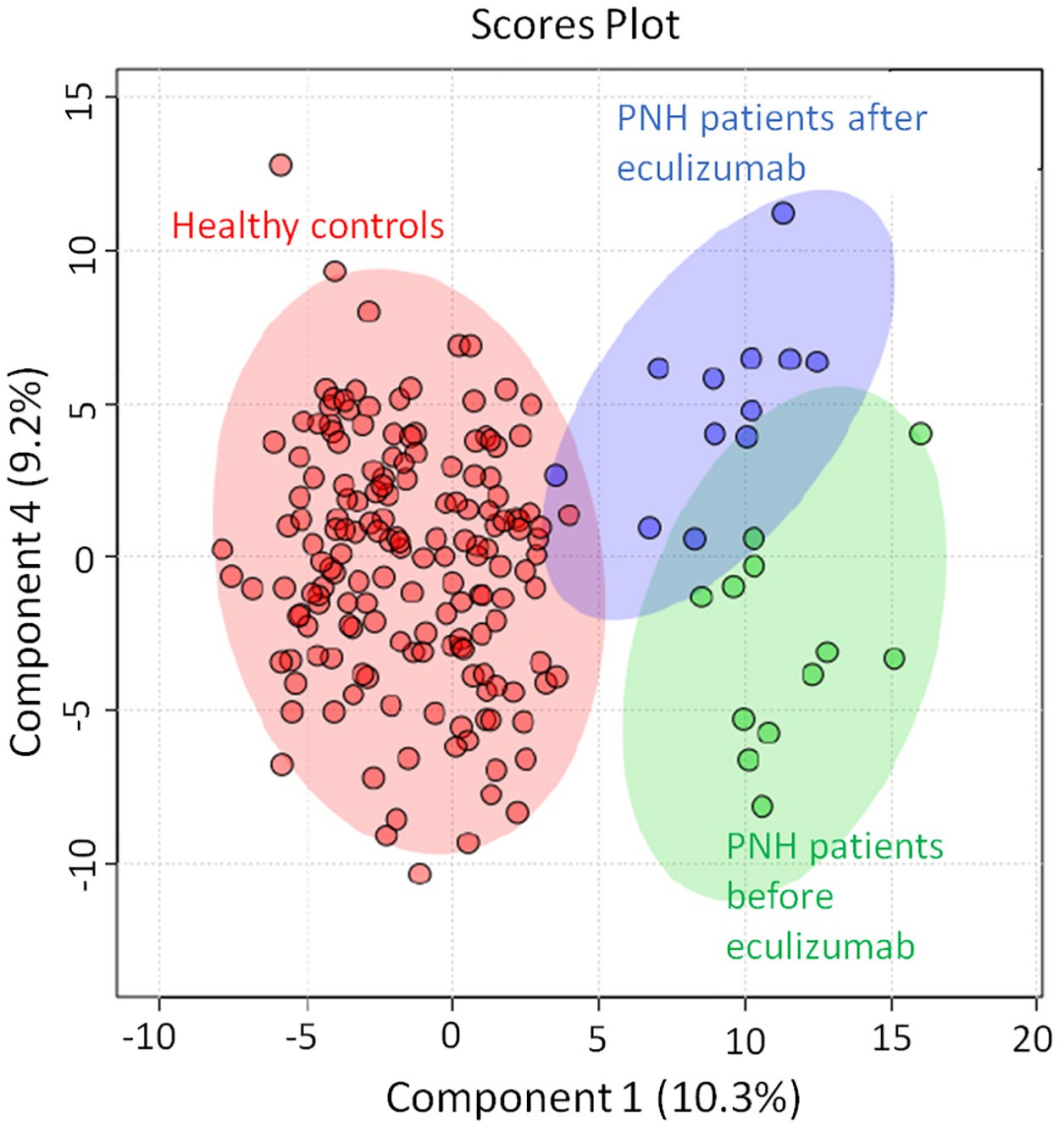
S-PLSDA multivariate analysis depicting a shift from the samples before (Green) to the samples after (Blue) eculizumab, and from that, towards healthy controls (Red). (Metaboanalyst 4.0 www.metaboanalyst.ca).

Biochemical phenotyping performed in the plasma samples collected immediately before and 24 hours after eculizumab infusion showed that inhibition of complement activation is followed by significant shifts towards normal values in the parameters previously revealing impairments in Acyl CoA metabolism (Fig 5).

**Fig 6:**
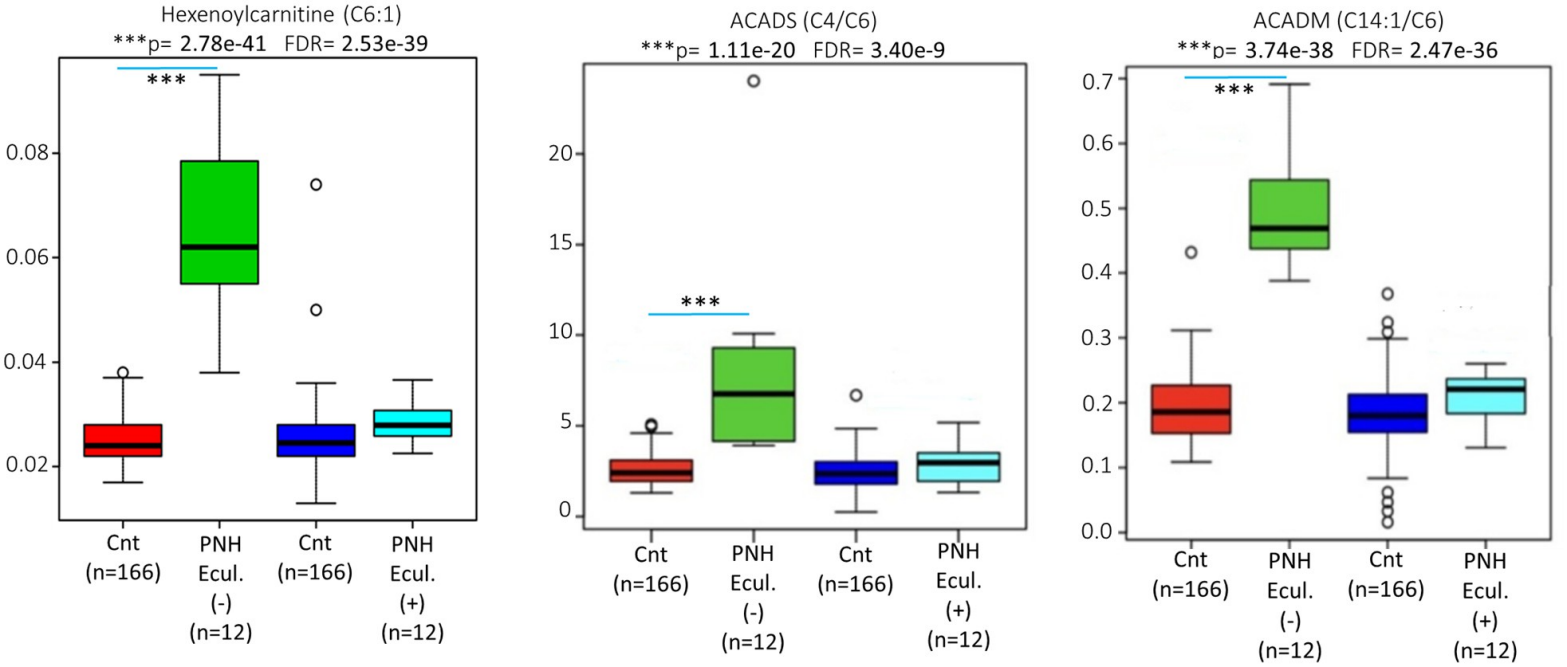
ANOVA statistical analysis of the C6:1 acylcarnitine concentrations, as well as the (C14:1/C6) and (C4/C6) ratios before (Green bar) and 24 hours after (Light blue bar) the use of eculizumab in the patients with PNH compared to the population-based controls (Cnt- Red and Dark blue bars).

Fig 6 shows the quantified validated metabolites in the blood samples from 12 patients collected 24 hours after eculizumab infusion (PNH-Ecul.+) compared to the metabolites in the samples collected from the same patients immediately before infusion (PNH-Ecul.-). Among these 12 PNH patients who were on regular complement inhibition by eculizumab, several altered phenotypes were observed: the concentrations of acylcarnitine C6:1, the C14:1/C6 ratio (reflecting the impaired action of medium-chain acyl-CoA dehydrogenase (ACADM), and the C4/C6 ratio (reflecting the impaired action of ACADS) were significantly increased prior to receiving eculizumab. Following the dose of eculizumab, 24 hours after drug administration, there was a significant decrease towards normalization of the above-mentioned metabolites.

**Fig 7:**
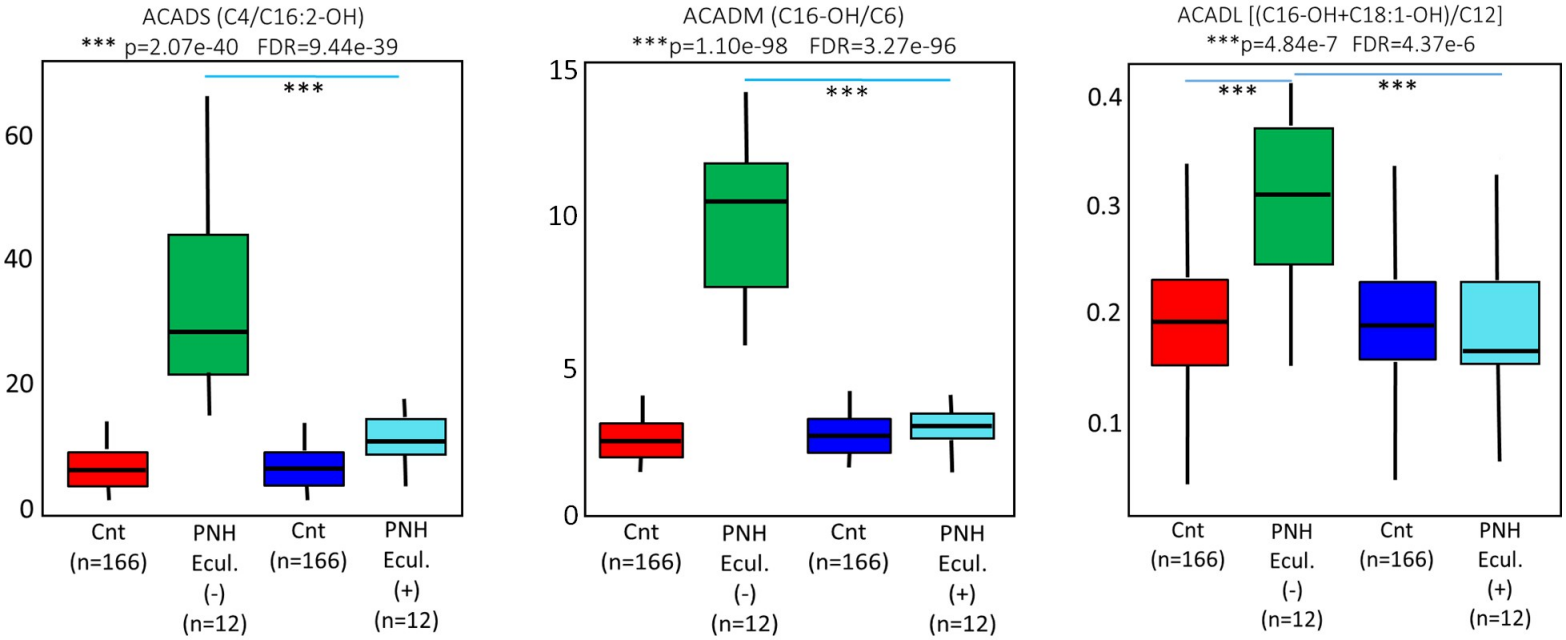
ANOVA statistical analysis before (Green bar) and 24h after (Light blue bar) the use of eculizumab in the PNH patients compared to population-based controls (cnt- Red and Dark blue bars). The results before eculizumab treatment, depicting Acyl-Coenzyme A Dehydrogenase Short Chain (ACADS), Acyl-Coenzyme A Dehydrogenase Medium Chain (ACADM), and Acyl-Coenzyme A Dehydrogenase Long Chain (ACADL) insufficiencies, are supported by the elevations of values generated by the ratios (C4/C16:2-OH), (C16-OH/C6), and [(C16-OH+C18:1-OH)/C12], respectively (Green bars). These results, however, were brought to levels closer to the controls 24 hours after eculizumab administration (Light blue bars).

**Fig 8:**
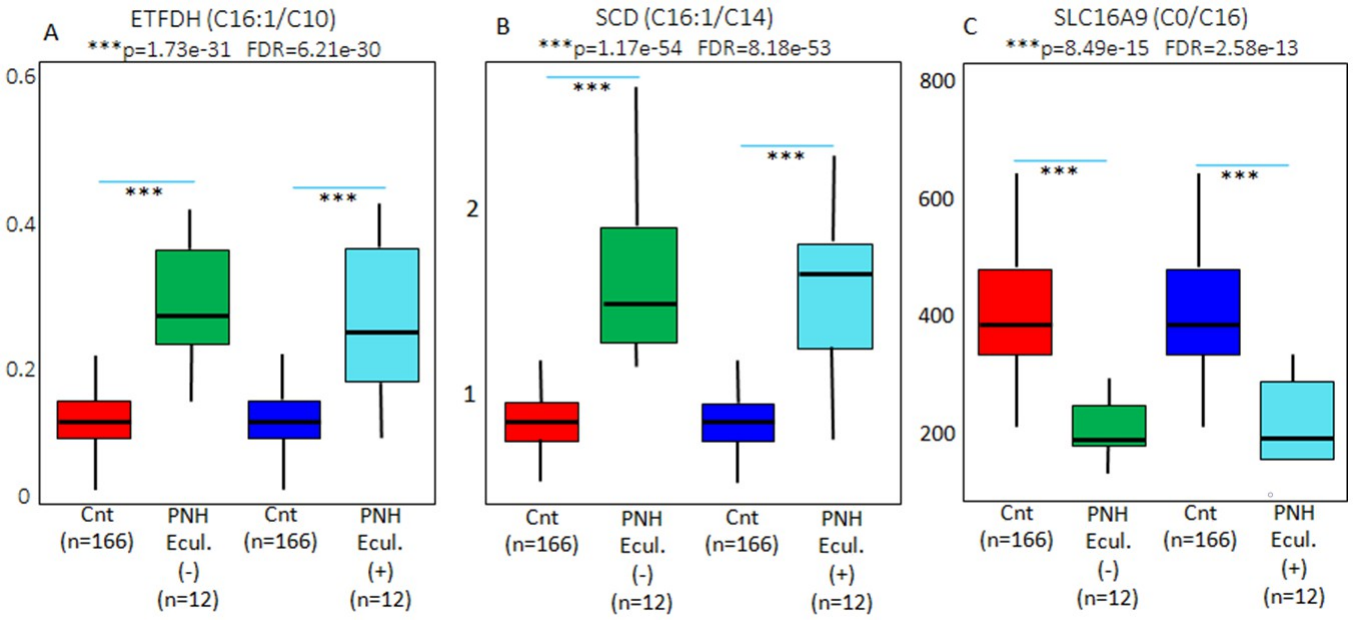
S-PLSDA multivariate analysis depicting a shift from the samples before (Green) to the samples after (Blue) eculizumab administration, and from that, towards the healthy controls (Red). The results before eculizumab treatment, depicting Electron-transferring Flavoprotein Dehydrogenase (ETFDH), Stearoyl Coenzyme A Desaturase (SCD), and Solute Carrier Family 16, member 9 (SLC16A9) insufficiencies, are supported by the values generated by the ratios (C16:1/C10), (C16:1/C14), and (C0/C16) respectively (Green bars). These results, different from acyl-coenzyme A dehydrogenases, were not brought to levels closer to the controls 24 hours after eculizumab administration (Light blue bars).

**Fig 9:**
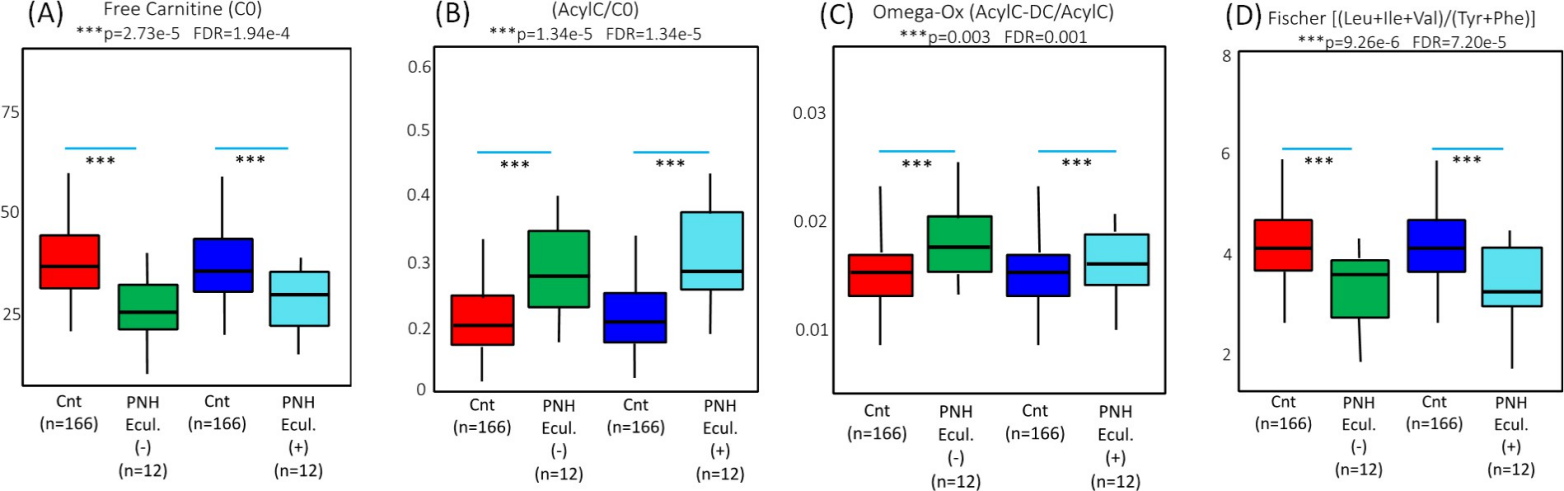
ANOVA results depicting values of the biochemical features before (Green) and 24h after (Light Blue) eculizumab administration. The results are suggestive of PNH as a condition of acyl CoA metabolism deficiency in which the concentrations of free L-carnitine (C0) are significantly low when compared to the controls (Fig 9A) and when assembled as the ratio AcylC/C0, the elevated values demonstrate accumulation in the blood of esterified acylcarnitine (AcylC) species which is comparable to mitochondrial β-oxidation deficiencies (Fig 9B). Further support implicating shortages in the mitochondrial function in PNH is the fact that, besides β-oxidation impairments, ω-oxidation was also found to be significantly (p = 0.003, FDR = 0.01) increased in the PNH cases as demonstrated in Fig 9C. Up-regulation of the liver microsomal ω-oxidation pathway, which is characterized by elevations in the ratio of total dicarboxylic (AcylC-DC) to total esterified acylcarnitines (AcylC) (Fig 9C), is considered a rescue pathway in situations where β and/or α-oxidation are decreased (Treem, 2011). From a hepatic function perspective, the liver is one of the most important organs to suffer the consequences of β-oxidation weakening; our results also demonstrate that PNH depicts biochemical signals of liver impairment (Fig 9D).

Indeed, eculizumab seems to exert a more evident effect against ACADM deficiencies, although short-chain acyl-CoA dehydrogenase (ACADS) and large-chain acyl-CoA dehydrogenase (ACADL) parameters were also affected (Fig 7). These findings demonstrate that the inhibition of complement activation may also bring mitochondrial dysfunctions closer to normality. Not all the metabolic ratios were equally affected following eculizumab infusion. Electron-transferring flavoprotein dehydrogenase (ETFDH), stearoyl coenzyme A desaturase (SCD), and solute carrier family 16, member 9 (SLC16A9) insufficiencies, different from the acyl-coenzyme A dehydrogenases, were not brought to levels closer to controls after 24 hours of eculizumab administration, as demonstrated in Fig 8. Moreover, ω-oxidation was also significantly increased (p = 0.003, FDR = 0.01, Fig 9) in the PNH cases, as demonstrated in Fig 9. These ratios and free carnitine levels were not brought to levels closer to the controls 24 hours after eculizumab infusion.

## Discussion

Some studies have described the metabolomic profile in hemolytic diseases and aplastic anemia. The main findings are summarized in Table 5. The first description on the differential metabolite cargo occurring in plasma exosomes of PNH patients, showed higher levels of prostaglandin F2-alpha. stearoyl arginine (5.3-fold), and 26-hydroxycholesterol-3-sulfate (11.2-fold) in the PNH patients compared to the healthy controls. Plasma exosomes can be a reservoir of (pro-inflammatory) circulating metabolites [22]. In the present study, we analyzed an extensive panel of plasma metabolites from patients with PNH using electrospray ionization tandem mass spectrometry. Patients with hemolytic PNH carry a distinct metabolomic profile involving both amino acid metabolism and the β-oxidation of fatty acids. We noted a significant increase in the plasma concentrations of long-chain acylcarnitines as well as a considerable reduction in free carnitine, mainly as a result of acyl-CoA dehydrogenases deficiencies. Eculizumab treatment seems to improve these deficiencies.

**Table 5:** Metabolomic studies in hemolytic anemia and aplastic anemia.

Disease	Main findings	Material	Reference
**PNH**	Reduction in amino acids participating in the glycogenesis pathway, impairing glutaminolysis. Low concentration of arginine Increase in polyamines resulting from the consequential increase in ornithine Free carnitine consumption and increased long-chain acylcarnitines	Plasma	Yamakawa PE *et al*
**Sickle cell- HbS**	Increase in intermediates of the glycolytic and pentose-phosphate pathways Increased malate levels Accumulation of the intermediates of ascorbate metabolism Decreased glutathione levels (involved in oxidative control) Accumulation of carnitines Increased amino acids (glutamine, glutamate, and glycine) Alteration of arginine and nitric oxide metabolism products	Erythrocytes	Darghouth D *et al*. *(2011)*.
**HbS / β thalassemia**	L-arginine and L-ornithine were significantly decreased Carnitine, acetylcarnitine, and propionylcarnitine correlated significantly with the reticulocyte production index (p <0.001)	Plasma	Papassotiriou *et al*. *(2013)*.
**β thalassemia**	Up-regulated: Fatty acid elongation in the mitochondria Glycolysis or gluconeogenesis, pyruvate metabolism, propanoate metabolism Glycerophospholipid metabolism Fatty acid biosynthesis, fatty acid metabolism Starch and sucrose metabolism Down-regulated: Glycerolipid metabolism, galactose metabolism, fatty acid biosynthesis, glyoxylate and dicarboxylate metabolism	Plasma	Musharraf SG *et al*. *(2017)*.
**β-thalassemia major**	Free carnitine, short- and long-chain acylcarnitines, and total acylcarnitine levels decreased Medium-chain acylcarnitines hexanoylcarnitine (C6) and decanoylcarnitine (C10) were high	Dry blood	Kumar S *et al*.*(2016)*.
**Autoimmune hemolytic anemia**	Increase in long-chain acylcarnitines, phosphatidylcholines, asymmetric dimethylarginine (ADMA), sphingomyelins, andpolyamines (spermine and spermidine)	Plasma	Rabelo IB *et al*. *(2018)*
**Aplastic anemia**	Higher levels of L-ornithine, L-proline, L-glutamine, L-tyrosine, creatinine, L-methionine, L-valine, L-leucine, L-phenylalanine, and L-tryptophan Lower levels of citric acid, succinic acid, and isocitric acid.	Plasma	Zhong P *et al*. *(2015)*.

These results suggest that PNH patients harbor impairments in acyl CoA metabolism, in particular, insufficiencies in the mitochondrial oxidative process of long and medium-chain fatty acids. Over time, as acyl CoA metabolism is further arrested, the available free L-carnitine is forced to rebound to acyl CoA species leading to a secondary carnitine deficiency with consequent impairments in β-oxidation due to an inability of the fatty acids to properly reach mitochondrial cytoplasm [32]. Consequently, the proportions of total acylcarnitine species to free L-carnitine are usually higher than values detected in the healthy population. In agreement to this possibility, in which the free L-carnitine (C0) levels are usually reduced [32], the mean plasma free L-carnitine concentration, in the PNH group, was significantly lower than in the control group.

Disturbances in the relative concentration of endogenous carnitines are associated with mitochondrial metabolism disorders responsible for energy generation through fatty acids. Fatty acid metabolism damage, such as deficiencies in short-, medium-, and long-chain acyl-CoA dehydrogenases (ACADS, ACADM, and ACADL), may lead to a decrease in their oxidation, increasing intermediary lipid metabolites, such as acylcarnitines.

Up-regulation in the liver microsomal ω-oxidation pathway, which is characterized by elevations in the ratio of total dicarboxylic (AcylC-DC) to total esterified acylcarnitines (AcylC), is regarded as a rescue pathway in situations where β or α-oxidations are decreased [33]. Further support implicating shortages in mitochondrial function in PNH is that, in addition to β-oxidation impairments, ω-oxidation was also found to be significantly increased in PNH cases. The identification of patients depicting lower levels of C0, as a single metabolite, might be related to dysfunctions in the Solute Carrier Transporter SLC16A9 [23, 30]. The association of SNPs present in SLC16A9, such as rs7094971, with free L-carnitine (C0) concentrations suggests that this compound could be a presumed substrate for this transporter, or, instead, that the substrate transported by this monocarboxylate transporter can directly influence the carnitine concentrations [23, 30].

The increase in acylcarnitines in PNH may also be related to the high erythrocyte cell membrane turnover. Red blood cells have been shown to have different carnitine compositions, with acylcarnitines comprising up to 52% of the total carnitine of erythrocytes compared to only 13% in plasma [34]. Thus, hemolysis can release more acylcarnitines from the erythrocytes into the plasma. Long-chain acylcarnitines are also capable of breaking down membrane barriers and producing complete membrane solubilization. Cho and Proulx [35] demonstrated lysis of human and rat erythrocytes in vitro after 15 minutes of incubation in DL-carnitine and choline synthetic fatty acyl esters. This finding raises the hypothesis that long-chain acylcarnitines interact with the hydrophobic bonds that form between lipids and proteins in erythrocyte cell membranes [36]. Other studies corroborating the relationship of acylcarnitines with hemolysis include patients with autoimmune hemolytic anemia [37] and showed an increase in some long- and medium-chain acylcarnitines, and two studies also included patients with sickle cell anemia [17, 19]. A reduction in free carnitine, increase in medium-chain acylcarnitines, and reduction in long and short chain acylcarnitines were also observed in patients with β-thalassemia, suggesting that there is less activity of CPT1 (carnitine palmitoyltransferase-1) in these patients but the maintenance of peroxisomal activity in the production of medium-chain acylcarnitines [38].

The reduction in free carnitine and the increase in the total concentrations of medium- and long-chain acylcarnitines in patients with PNH indicate that the balance between different acylcarnitines is impaired and may contribute to symptoms related to weakness, pulmonary hypertension, and cardiovascular events. This pattern resembles biochemical events described in inborn errors of systemic metabolism, particularly dysfunctions of mitochondrial β-oxidation and peroxisome. The results were similar to those observed in the deficiencies of medium- and long-chain acyl-CoA dehydrogenases and deficiencies in free carnitine transport. The increase in acylcarnitines in the PNH patients in our study may also illustrate their interaction with the erythrocyte membrane favoring hemolysis. Thus, a better understanding of the altered acylcarnitine balance in patients with PNH requires further study.

The C14:1/C16:1 acylcarnitine (tetradecenoylcarnitine / palmitoleylcarnitine) ratio possibly suggests that lipid elongation is facilitated by and may be linked to increased desaturase-9 activity. When we evaluated the patients immediately prior to receiving their eculizumab dose and 24 hours after drug administration, there was a significant decrease towards normalization in the C6:1 levels, as well as in the C14:1/C6 (impaired MCAD activity) and C4/C6 (impaired SCAD activity) ratios, which were very high before drug administration. However, all the PNH patients on eculizumab were stable in the maintenance phase with good intravascular hemolysis control for several months when the samples were collected. We speculate whether these findings might be explained by a less stable and less sustained complement inhibition of eculizumab, determined by less stable free C5 levels [39], as well as a tendency to higher risk of breakthrough hemolysis of eculizumab as demonstrated in a phase 3 open-label study comparing eculizumab to ravulizumab [40]. Besides, most of the patients still presented with anemia and reticulocytosis, probably due to persistent extravascular hemolysis which can at least partly explain the metabolomic findings.

We found some reduced phosphatidylcholines and some increased lysophosphatidylcholines in the patients with PNH, indicating that these patients also have a disorder in the metabolism of glycerophospholipids. This finding might be related to the impaired metabolism of fatty acids and possibly contributes to the endothelial dysfunction observed in these patients. In addition, phosphatidylcholines serve as reservoirs of fatty acids (arachidonic acid) that are precursors of lipid-derived signaling molecules. Hydrolysis of a phosphatidylcholine molecule results in the production of lysophosphatidylcholine, which is a bioactive lipid involved in monocyte recruitment, vascular smooth muscle cell proliferation, and endothelial dysfunction [36].

In patients with PNH, the finding of low levels of glutamate, glutamine, alanine, and aspartate may indicate that glutaminolysis is impaired in this clinical condition. The reduced concentrations of these amino acids may also be linked to the activities of the plasma glutamate and glutamine transport systems. In patients with sickle cell anemia, increased concentrations of glutamate, glutamine, glycine, and alanine in sickle cell preparation have been reported, suggesting increased plasma glycine levels and increased glutamine and glutamate transport system activities [17].^.^

We also observed a decrease in the plasma concentrations of several amino acids: arginine, histidine, methionine, valine, serine, alanine, and aspartate. All of these amino acids can be transformed into pyruvate or a Krebs cycle intermediate to generate glucose, suggesting that there may be an impairment in the glycogenesis of these patients. Asparagine was the only amino acid that increased in this glycogenic pathway. The amino acids involved in the ketogenic pathway were not significantly altered.

Hemolysis causes high levels of cell-free hemoglobin which results in the consumption of endogenous NO and also releases erythrocyte arginase 1, an enzyme that converts L-arginine, the substrate for NO synthesis and reactive nitrogen species, to ornithine. This conversion depletes the plasma pool of arginine and reduces the systemic availability of NO [41]. Nitric oxide has a vasodilatory action and NO deficiency plays an important part in the mechanisms involved in smooth muscle dystonias in PNH, causing dysphagia, abdominal pain, erectile dysfunction, and other effects which have a very significant impact on quality of life [3].

In our study, we found reduced arginine and increased ornithine in patients with PNH, suggesting increased arginase activity. The conversion of arginine to ornithine and urea by arginase mainly occurs in the liver, but arginase 1 activity is particularly abundant in red blood cells and reticulocytes [41]. Increased arginase activity is seen in sickle cell patients as a consequence of inflammation, hepatic dysfunction, and mainly, by the release of erythrocyte arginase during hemolysis [42]. Arginine deficiency in sickle cell patients is associated with elevated arginase levels and a low arginine-ornithine ratio that correlates with markers of hemolysis [43]. Hill et al. [8] described association between intravascular hemolysis and NO depletion through the direct binding to free hemoglobin and reduction of NO production through reduced arginase 1 availability in patients with PNH.

The degradation of arginine by erythrocyte arginase generates ornithine, a metabolite that is a precursor for the synthesis of polyamines, and when degraded, it generates putrescine, and later, spermine and spermidine. Compared to the controls, the patients with PNH had increased spermidine concentrations, indicating increased catabolism of ornithine, which is also observed in patients with sickle cell anemia [42] and autoimmune hemolytic anemia [37].

Chronic hemolysis, the proinflammatory state and oxidative stress observed in patients with PNH may be associated with the metabolic findings found in this study. The low concentrations of phosphatidylcholine, taurine, and histidine, may increase oxidative stress and facilitate vascular events, which are additional risk factors for this group of patients. There are studies that suggest a relationship between the reduction in serum taurine and the progression of atherosclerosis and coronary vascular disease and that its presence is a protective factor against heart failure and coronary ischemia [44, 45]. A low plasma concentration of histidine was also associated with cardiovascular disease, heart failure, the presence of atherosclerosis, conditions related to inflammation, and oxidative stress [46]. In a registry of 2356 PNH patients, vascular events were the third major cause of mortality, accounting for 11% of deaths [47].

Our study had several limitations: the relatively small number of patients, which could be anticipated due to the rarity of the disease, and the fact that samples from PNH patients who were on eculizumab were obtained during maintenance phase, and not before the start of treatment. Indeed, all the PNH patients on eculizumab were stable in the maintenance phase with excellent control over intravascular hemolysis when samples were collected. Unfortunately, we could not evaluate the metabolomic profile of the patients before the start of eculizumab therapy. Most of the patients still had anemia and reticulocytosis, probably due to extravascular hemolysis, which can explain the metabolomic findings related to hemolysis. More studies, investigating a larger number of hemolytic PNH patients at diagnosis, before treatment, during eculizumab treatment, and at different time points after infusion, are required to better understand these findings. Studies performing plasma metabolites in other groups of patients with other hemolytic anemias can also provide further evidence of shared mechanisms.

In addition, we found an unpredicted effect of eculizumab infusion in improving deficiencies in Acyl CoA metabolism. Treatment with eculizumab may have a role in the mitochondrial oxidative process of long and medium-chain fatty acids,reducing oxidative stress and inflammation, and bringing acylcarnitine concentrations close to normal levels.

In conclusion, we found a unique metabolomic profile in the PNH patients; this profile was characterized by a reduction in amino acids participating in the glycogenesis pathway, impairing glutaminolysis and an altered acylcarnitine balance. The profile of a few other metabolites in PNH was similar to that observed in other hemolytic disorder: a low concentration of arginine, which serves as a substrate in the biosynthesis pathway of nitric oxide, an increase in polyamines, resulting from the consequential increase in ornithine, and disturbances in carnitine and acylcarnitine homeostasis, with free carnitine consumption and increased long-chain acylcarnitines. Some of these differences seemed to be significantly reduced with eculizumab therapy, demonstrating that blocking complement can also improve the metabolic dysfunction of these patients. The distinct metabolomic profile of PNH patients can be of interest in the future for evaluating responses and monitoring the effects of novel treatment strategies.
